# Income Inequality and Adolescent Gambling Severity: Findings from a Large-Scale Italian Representative Survey

**DOI:** 10.3389/fpsyg.2017.01318

**Published:** 2017-08-03

**Authors:** Natale Canale, Alessio Vieno, Michela Lenzi, Mark D. Griffiths, Alberto Borraccino, Giacomo Lazzeri, Patrizia Lemma, Luca Scacchi, Massimo Santinello

**Affiliations:** ^1^Department of Developmental and Social Psychology, University of Padova Padova, Italy; ^2^International Gaming Research Unit, Psychology Department, Nottingham Trent University Nottingham, United Kingdom; ^3^Department of Public Health and Paediatrics, University of Turin Turin, Italy; ^4^Department of Molecular and Developmental Medicine, CREPS University of Siena – AOUS Siena, Italy; ^5^Department of Human and Social Sciences, University of Valle d'Aosta Aosta, Italy

**Keywords:** gambling, adolescent gambling, youth gambling, problem gambling, inequality, representative survey

## Abstract

**Background:** Studies have shown that problems related to adult gambling have a geographical and social gradient. For instance, adults experiencing gambling-related harms live in areas of greater deprivation; are unemployed, and have lower income. However, little is known about the impact of socioeconomic inequalities on adolescent problem gambling. The main purpose of the present study was to investigate the contextual influences of income inequality on at-risk or problem gambling (ARPG) in a large-scale nationally representative sample of Italian adolescents. A secondary aim was to analyze the association between perceived social support (from family, peers, teachers, and classmates) and ARPG.

**Methods:** Data from the 2013–2014 Health Behavior in School-aged Children Survey (HBSC) Study was used for cross-sectional analyses of ARPG. A total of 20,791 15-year-old students completed self-administered questionnaires. Region-level data on income inequality (GINI index) and overall wealth (GDP per capita) were retrieved from the National Institute of Statistics (Istat). The data were analyzed using the multi-level logistic regression analysis, with students at the first level and regions at the second level.

**Results:** The study demonstrated a North–South gradient for the prevalence of ARPG, with higher prevalence of ARPG in the Southern/Islands/Central Regions (e.g., 11% in Sicily) than in Northern Italy (e.g., 2% in Aosta Valley). Students in regions of high-income inequality were significantly more likely than those in regions of low-income inequality to be at-risk or problem gamblers (following adjustment for sex, family structure, family affluence, perceived social support, and regionale wealth). Additionally, perceived social support from parents and teachers were negatively related to ARPG.

**Conclusions:** Income inequality may have a contextual influence on ARPG. More specifically, living in regions of highest income inequality appeared to be a potential factor that increases the likelihood of becoming an at-risk or problem gambler. Findings of the study suggest that wealth distribution within societies affected by economic policies may indirectly have an influence adolescent gambling behaviors.

## Introduction

Gambling disorder is a recognized mental health condition that comprises persistent and recurrent problem gambling causing individuals significant psychological impairment and/or distress (American Psychiatric Association, 2013). Furthermore, in many countries, problem gambling among adolescents has emerged as an increasing social and public health issue (Volberg et al., 2010; Molinaro et al., 2014; Calado et al., 2016) including the country of the present study (i.e., Italy). Despite Italian legislation prohibiting minors from participating in legalized gambling, previous research has shown that youth gambling is a popular activity in Italy. For instance, results from the European-School-Survey-Project-on-Alcohol-and-Other-Drugs (ESPAD-Italia®), conducted annually on representative sample of students 15–19 year-olds, showed that during 2012, 18% of students gambled at least once a month on one or two gambling activities (frequent gamblers; Canale et al., 2016b) and during 2013, 6.5% were classified as problem gamblers (Canale et al., 2016a).

Additionally, an analysis of the ESPAD-Italia®2011 data showed that the prevalence of at-risk/problem gambling was higher for adolescents living in more disadvantaged regions (Gori et al., 2015), suggesting that problem gambling can also be considered a social problem (Reith and Dobbie, 2011). Although, Gori et al. (2015) used socio-cultural indicators such as the (i) unemployment rate, (ii) non-engagement rate in Education, Employment or Training (NEET), and (iii) part of per capita GDP expended in gambling activities, it did not focus on structural determinants of adolescent health, for example national wealth or income inequality (e.g., Dorling et al., 2007; Viner et al., 2012). Recently, socioeconomic inequality has shown to have an increasing impact on adolescent health (e.g., Elgar et al., 2015). Additionally, structural determinants of adolescent health (e.g., health expenditure) have been associated with lower levels of probable gambling problems among representative samples of students living in nine European countries (Albania, Cyprus, Denmark, Finland, Italy, Lithuania, Romania, Serbia, and the United Kingdom; Molinaro et al., 2014). However, to date, no studies have investigated the association between structural determinants of adolescent health, such as socioeconomic inequality, and adolescent problem gambling in general, and specifically in Italy, a country characterized by large and rising levels of inequality and poverty. Over the last thirty years, Italy has seen an increase in income inequalities. According to the 2015 Luxembourg Income Study, Italy registered some increase (0.05 to 0.1 point per year) in the Gini Index from 1980 to 2010 (Thewissen et al., 2015). The levels of income inequality have been magnified by the economic crisis and are reflected by regional differences, with the more disadvantaged areas also being the more unequal (The World Top Incomes Database, 2011). Thus, the present study examined the association between structural determinants of adolescent health (i.e., regional income inequality and GDP) and problem gambling in Italy, a country characterized by rising levels of inequality and poverty (Thewissen et al., 2015).

Problem gambling is governed by a complex set of interrelating factors, causes, and determinants ranging from biology and family history to social norms and existing statutes (Messerlian and Derevensky, 2005; Abbott et al., 2013). Consequently, many factors may come into play in various ways and at different levels that together contribute to the development and maintenance of gambling-related problems (e.g., biological, psychological, or social). According to the conceptual framework for the development of gambling in youth (Barnes et al., 1999) and the conceptual framework of harmful gambling (Abbott et al., 2013), broader perspectives are important when considering problem gambling, including both contextual macro-level factors (social, economic, and political forces) and interpersonal factors (e.g., support from parents, friends). Thus, the present study provides new insight into the possible combination of interpersonal and macro-level factors in explaining the development of adolescent gambling severity.

### Socioeconomic inequalities in adolescent health: the case of adolescent gambling

Within contextual factors, there is an association between societal inequality and many different negative health and social outcomes, such as sexual promiscuity, teenage pregnancy, violence, substance abuse, crime, psychological and physical disorders, and life satisfaction (see Pickett and Wilkinson, 2015 for a recent review; Elgar et al., 2015). On the other hand, the distribution of gambling problems reflects the geographic distribution of socioeconomic deprivation (Reith, 2012). For instance, in Australia, the greater the socioeconomic disadvantage of a municipality, the higher its numbers of gambling opportunities (e.g., gaming machines), with people living in areas of high deprivation spending close to twice as much the state's mean expenditure on slot machines (Livingstone, 2001). Problem gambling also has a social and geographical gradient. For instance, adults experiencing gambling-related harm (i) live in areas of greater deprivation, (ii) are unemployed, and (iii) have lower income (Orford et al., 2010; Wardle et al., 2014). A growing body of laboratory studies suggests that people who feel relatively deprived have more severe gambling problems (Callan et al., 2008; Haisley et al., 2008; Mishra and Novakowski, 2016; Tabri et al., 2017). Consistent with the risk-sensitivity theory (Caraco et al., 1980), victims of income inequality engage in greater risk-taking behaviors (Mishra et al., 2014) because inequality facilitates the perception of need in that victims of inequality are at distance from the desired or goal state or more privileged others.

Although there is great empirical evidence of an inequality-risk association at the societal level, unexpectedly little research has studied whether inequality at societal level is associated with adolescent gambling. Inequality, more specifically income inequality, might be associated with adolescent gambling because income inequality is responsible for an intensification of societal class competition, that when compared to more egalitarian societies, makes status increasingly important for survival (Wilkinson, 2004; Wilkinson and Pickett, 2009). Status competition in more hierarchical societies increases because greater numbers of people are deprived access to success and status markers (Wilkinson and Pickett, 2009). Among minors, who are acutely aware of class differences, inequality in income might increase the social distance between such individuals who live in the same society fostering a tough social environment that regularly features acts of rejection, teasing, and humiliation (Elgar et al., 2009).

Income inequality intensifies perceptions that an individual is unjustly resource disadvantaged relative to others. Such relative deprivation is accompanied by feelings of anger and resentment (Crosby, 1976; Smith et al., 2012) that motivates a desire to move up the social ladder, especially individuals lower down the income distribution (Wilkinson and Pickett, 2009). Thus, it possible that when individuals perceive themselves as unfairly deprived they may also engage in maladaptive behaviors to advance their financial position. For example, individuals who feel relatively deprived are apt to gamble in an attempt to quickly reduce their perceived financial disadvantage (see Callan et al., 2015 for a meta-analysis). More recently, Tabri et al. (2017) showed that relative deprivation is most likely to lead to disordered gambling when individuals perceived a low personal capacity for upward economic mobility via conventional means (e.g., professional development activities). In this context, gambling may be considered by adolescents as a means: (i) to help people meet their needs and wants and/or offset feelings of deprivation through the possibility of financial windfall (Mishra et al., 2017); and/or (ii) to be a path to upward economic mobility (Tabri et al., 2015). Thus, the principal aim of the present study was to verify the association between income inequality and adolescent problem gambling at a societal level (regions).

### Social support and gambling

Social support has been cited as a protective factor against a wide range of risk behaviors, including adolescent problem gambling. Social support provided by individuals and institutions is defined as interpersonal relationships that are able to influence the way in which individuals live. Supportive relationships with others (i.e., social support) have been conceptualized as resources that promote successful adaptation during adolescence (Compas et al., 1995; Juang and Silbereisen, 1999; Saunders et al., 2004; Moor et al., 2015). During adolescence, there are several potential sources of social support (e.g., parents, siblings, friends, classmates, and teachers) and they are sensitive to the interconnections between these sources (Benhorin and McMahon, 2008). Examples of social support include parent's closeness, monitoring and caring, teachers' interest in their students, and friends' supportiveness (Jessor et al., 2003). Perceived social support is frequently used in the study of adolescent development as a proxy for good social support (Wills and Shinar, 2000). Such support denotes the perceived extent by people to which individuals within their social networks can provide social support (Demaray and Malecki, 2002). High social support may protect against gambling-related harms by promoting social environments whereby adolescents feel accepted and wanted without teasing, rejection, and humiliation related to social comparisons (e.g., reducing status competition within society).

Previous studies have found that social support from school, parents, and friends all influence adolescent problem gambling. For example, non-gamblers and social gamblers perceive they have more social support from parents and friends (e.g., having parents and peers who provided support and encouragement) than at-risk and problem gamblers (Hardoon et al., 2004; Molinaro et al., 2014; Canale et al., 2017). Similarly, high forms of social support from school and teachers have shown to be protective against gambling participation among 14–16-year-old Finnish adolescents (Räsänen et al., 2016). More specifically, it was found that having teachers who provided support and encouragement within a supporting schools setting (e.g., schools helping students when they need it) reduced the odds of being engaged in gambling activities (Räsänen et al., 2016). It appears that supportive families, supportive schools, together with supportive peers, are crucial in protecting adolescents from gambling-related harms. These social relationships, that have been found to differ between the most and least unequal regions (De Clercq et al., 2016; Ng Fat et al., 2016), might also moderate the influence of inequality act on adolescent gambling severity. Indeed, the lack of social support might exacerbate the impact of income inequality on adolescent problem gambling. Thus, the present study intended to clarify the additive role of social support and macro-level factors related to adolescent gambling severity.

### The present study

Consistent with the literature reviewed, the principal aim of the present study was to establish the relationships between at-risk or problem gambling (ARPG) and income inequality. It is hypothesized that in regions with higher levels of income inequalities, adolescents would report higher levels of ARPG compared to more egalitarian regions. A secondary aim was to analyze the association between perceived social support (from family, peers, teachers, and classmates) and ARPG. It was also hypothesized that adolescents who perceived more social support would be less likely to report higher levels of ARPG than those who perceived less social support. Finally, another aim of the present study was to explore possible interactions between perceived social support and region-level inequality in influencing problem gambling. It was hypothesized that the impact of living in a more unequal region on ARPG would be stronger for adolescents perceiving lower levels of social support.

## Methods

### Participants

The data were collected in the Italian 2013–2014 Health Behavior in School-aged Children (HBSC) survey. An aim of the HBSC study (see http://www.hbsc.org for more details) was to identify behaviors and social factors that influence behavioral addictions (including ARPG) in youth. In Italy, students from Grade 6 (11-year olds) to Grade 10 (15-year olds) secondary schools were invited to participate. Because assessments of gambling were only included in the 15-year-olds'questionnaires, 11- and 13-year-old students were excluded from the present study. The sample comprised 20,791 students (male, 50.3%) nested within 1,050 schools and 21 Italian regions/cities (Abruzzo, Aosta Valley, Basilicata, Calabria, Campania, Emilia-Romagna, Friuli Venezia Giulia, Latium, Liguria, Lombardy, Marches, Molise, Piedmont, Puglia, Sicily, Sardinia, Trentino, Tuscany, Umbria, and Veneto)^1^. A random sample of schools was drawn from the National School Office. The average participation rate by students was 91%. Nationally representative samples of students in Grade 10 participated in the present study. The self-completion questionnaires were administered by classroom teachers during normal school day classes. The questionnaire took ~50 min to complete. Written informed consent was obtained from the parents of the students of this study and all participants were assured of the confidentiality of their responses. The University of Turin's Ethics Committee granted ethical approval for the study.

### Measures

The current study comprises a secondary data analysis of the Italian HBSC 2013–2014 survey which includes questions related to a number of different behaviors. The reliability and validity of these scales assessing such behaviors among teenagers in various countries is well-established (Lazzeri et al., 2013).

#### Dependent variable

At-risk or problem gambling (ARPG) was assessed with the 12-item South Oaks Gambling Screen-Revised for Adolescents SOGS-RA (Italian version: Chiesi et al., 2013). Participants were presented with 12 items assessing negative consequences associated with gambling behavior over a past-year timeframe on a binary “yes-no” scale scored 1 or 0, respectively. The original scoring system of Winters et al. (1995) was used to estimate prevalence rates of ARPG during the past 12 months. The scoring was as follows: 0–1 = “no gambling problem,” 2–3 = “at-risk gambling,” and 4 or more = “problem gambling.” In previous studies, ARPG has been considered as part of a wider spectrum of problematic adolescent gambling (Potenza et al., 2011). Consistent with previously used groups, they were dichotomized into “at-risk-problematic gamblers” and “non-problematic gamblers” (Wickwire et al., 2007; Potenza et al., 2011). The instrument had adequate internal reliability (α = 0.78; 95% CI = 0.78–0.80). In a recent systematic review, Edgren et al. (2016) found that most studies examining adolescent gambling used the SOGS-RA as the primary ARPG instrument. In addition, items on frequency of gambling involvement (in their lifetime and in the last 30 days) were also included, as well as the number of gambling occasions (“During the last 30 days/In your lifetime, on how many occasions [if any] have you participated in gambling activities?”—seven options ranging from “never” to “30 or more days”). A binary variable was created describing the gambling lifetime frequency (0 = never; 1 = from “1–2 days” to “30 or more days”).

#### Individual-level variables

##### Family structure

Family structure was assessed utilizing responses to the single question “Which of the following people live in the same household with you?” to indicate students who lived with two biological or adoptive parents or those that lived in other types of family set-up (e.g., single-parent families; Hamilton et al., 2014).

##### Family wealth

Family wealth was assessed using the Family Affluence Scale (FAS) (Boyce et al., 2006). The FAS refers to familial (material) wealth by asking questions relating to number of family holidays over the past 12 months, the number of household cars, the number of home computers in the house, and whether participants had a bedroom of their own. Scores ranged from zero to seven and were divided into three groups. Students scoring between zero and four were placed into the “low-affluence” category, those scoring between five and six were placed into the “moderate-affluence” group, and those who scored seven were placed in the “high-affluence” category. Previous studies indicated that compared to other family affluence measures relying on parental occupation, education and/or income, the FAS has superior criterion validity and is much less affected by nonresponse bias (Boyce et al., 2006; Currie et al., 2008). Social support was measured using a perceived support definition of social support with the following four sources of support: teachers, classmates, parents, and friends.

##### Perceived classmate support

Perceived classmate support was assessed with three items from the Teacher and Classmate Support Scale (Torsheim et al., 2000): “The students in my class enjoy being together,” “Most of the students in my class are kind and helpful,” and “Other students accept me as I am.” Items were rated on a 5-point frequency scale from (1) “strongly agree” to (5) “strongly disagree.” Responses were reverse coded and then the three items were averaged. Higher scores indicate a higher level of perceived support from classmates. Alpha reliability for the 3-item scale was 0.76 (95% CI = 0.75–0.77).

##### Perceived teacher support

Perceived teacher support was assessed using three items: “I feel my teacher accepts me as I am,” “I feel that my teachers care about me as a person” and “I feel a lot of trust in my teachers” (e.g., Klemera et al., 2016; Bjereld et al., 2017). Alpha reliability for the 3-item scale was 0.79 (95% CI = 0.78–0.80). Higher scores indicate a higher level of perceived support from teachers.

##### Perceived friend support

Perceived friend support was assessed using four items from a sub-scale of the Multidimensional Scale of Perceived Social Support (Zimet et al., 1988): “My friends really try to help me,” “I can count on my friends when things go wrong,” “I have friends with whom I can share my joys and sorrows” and “I can talk about my problems with my friends.” Items were rated on a 7-point frequency scale from (1) “strongly disagree” to (7) “strongly disagree.” Alpha reliability for the 4-item scale was 0.90 (95% CI = 0.89–0.91). Responses were averaged in order to assess perceived friend support.

##### Perceived family support

Perceived family support was assessed by four items from a sub-scale of the Multidimensional Scale of Perceived Social Support (Zimet et al., 1988): “My family really tries to help me,” “I get the emotional help and support I need from my family,” “I can talk about my problems with my family” and “My family is willing to help me make decisions.” Items were rated on a 7-point frequency scale from (1) “strongly disagree” to (7) “strongly disagree.” Alpha reliability for the 4-item scale was 0.89 (95% CI = 0.89–0.90). Responses were averaged in order to assess perceived family support.

#### Regional-level variables

Data on Italian regional wealth (gross domestic product [GDP] per capita) and income inequality (Gini index) were taken from the National Institute of Statistics (ISTAT, see www.istat.it). These data are presented in Table 1. The Gini index denotes the distribution of income or consumption among citizens in a society, and ranges theoretically from 0 (where all persons have equal income; perfect equality) to 1 (where one person has all the income and the rest have none; perfect inequality). With the aim of facilitating logistic regression analysis, regions were grouped into approximate thirds of low, medium, and high income inequality based on Gini indices (e.g., Elgar et al., 2005), as presented in Table 1.

**Table 1:** Descriptive statistics for the Italian regional variables: Data provided for regions/cities (*n* = 21)^#^.

**Country**	***n***	**Gini index**	**GDP per capita**	**At-risk or problem gambling % (n)**					**Gambling frequency%(*****n*****)—lifetime prevalence**				
				**Total**	**Boys**	**Girls**	**χ^2^**	**Φ**	**Total**	**Boys**	**Girls**	**χ^2^**	**Φ**
**LOW INCOME INEQUALITY**													
Aosta Valley	521	0.246	37.00	2.0 (11)	4.0 (8)	1.0 (3)	3.93^*^	0.09	29.0 (150)	46.0 (105)	15.0 (45)	57.65^***^	0.33
Bolzano	729	0.260	39.90	2.0 (13)	3.0 (10)	1.0 (3)	6.9^**^	0.10	41.0 (299)	48.0 (145)	36.0 (154)	10.86^**^	0.12
Friuli Venezia Giulia	1,005	0.260	27.90	5.0 (46)	7.0 (37)	2.0 (9)	11.44^**^	0.11	33.0 (331)	44.0 (248)	19.0 (83)	73.02^***^	0.27
Trento	796	0.270	33.90	2.0 (16)	3.0 (12)	1.0 (4)	4.00^*^	0.07	28.0 (224)	36.0 (144)	20.0 (80)	25.95^***^	0.18
Veneto	2,447	0.270	30.00	4.0 (89)	6.0 (63)	2.0 (26)	17.68^***^	0.09	30.5 (744)	45.0 (530)	17.0 (214)	224.59^***^	0.30
**MEDIUM INCOME INEQUALITY**													
Abruzzo	737	0.300	23.10	11.0 (75)	18.0 (64)	3.0 (11)	39.95^***^	0.24	44.0 (321)	66.0 (245)	21.0 (76)	150.68^***^	0.46
Basilicata	576	0.280	18.70	9.0 (43)	16.0 (39)	2.0 (4)	32.23^***^	0.25	41.0 (231)	63.0 (176)	19.0 (55)	117.79^***^	0.45
Emilia-Romagna	1,116	0.290	32.50	6.0 (64)	10.0 (56)	2.0 (8)	30.22^***^	0.17	35.0 (390)	48.5 (293)	19.0 (97)	106.02^***^	0.31
Lombardy	1,474	0.300	35.00	5.0 (75)	9.0 (65)	2.0 (10)	37.01^***^	0.16	44.0 (653)	58.0 (445)	30.0 (208)	117.00^***^	0.29
Marche	978	0.280	25.20	5.0 (50)	11.0 (43)	1.0 (7)	41.48^***^	0.21	39.0 (379)	58.0 (241)	25.0 (138)	112.85^***^	0.34
Molise	845	0.290	20.30	9.0 (71)	15.0 (61)	3.0 (10)	34.99^***^	0.21	38.0 (319)	59.0 (262)	14.0 (57)	174.55^***^	0.45
Piedmont	1,004	0.286	27.80	6.0 (53)	9.0 (43)	2.0 (10)	21.70^***^	0.15	33.0 (332)	49.0 (242)	18.0 (90)	108.79^***^	0.33
Puglia	1,070	0.310	16.90	9.0 (89)	15.0 (77)	2.0 (12)	47.42^***^	0.22	40.0 (426)	56.0 (306)	23.0 (120)	119.80^***^	0.34
Tuscany	1,030	0.280	28.90	3.0 (26)	5.0 (23)	1 (3)	17.84^***^	0.14	32.0 (331)	49.0 (240)	17.0 (91)	115.07^***^	0.34
Umbria	1,065	0.300	23.90	5.0 (46)	7.0 (40)	1.0 (6)	20.63^***^	0.14	38.0 (404)	56.0 (319)	17.0 (85)	167.70^***^	0.40
**HIGH INCOME INEQUALITY**													
Latium	955	0.350	31.70	7.0 (63)	11.0 (56)	2.0 (7)	33.95^***^	0.19	41.0 (387)	56.0 (285)	23.0 (102)	112.17^***^	0.34
Liguria	1,087	0.320	29.00	5.0 (51)	8.0 (40)	2.0 (11)	19.26^***^	0.14	33.0 (355)	48.0 (253)	18.0 (102)	106.37^***^	0.31
Campania	841	0.350	16.80	9.0 (72)	14.0 (60)	3.0 (12)	25.74^***^	0.18	48.0 (400)	72.0 (328)	19.0 (72)	240.60^***^	0.54
Calabria	883	0.320	16.20	10.0 (81)	16.0 (68)	3.0 (13)	37.70^***^	0.22	46.0 (404)	65.0 (299)	25.0 (105)	137.53^***^	0.40
Sicily	843	0.360	17.00	11.0 (84)	19.0 (77)	2.0 (7)	59.02^***^	0.28	43.5 (365)	66.0 (288)	19.0 (77)	189.44^***^	0.48
Sardinia	789	0.320	19.80	6.0 (40)	9.0 (32)	2.0 (8)	17.51^**^	0.15	33.0 (261)	51.0 (194)	17.0 (67)	104.02^***^	0.37

*** *p < 0.001*;

** *p < 0.01*;

* *p < 0.05*;

# *The number of regional levels totals 21 because the Trentino data comprised two different geographic areas (i.e., Bolzano and Trento)*.

### Data analysis

Prevalence of ARPG was compared by gender using a χ^2^ test. For the χ^2^ test, the phi (Φ) coefficient is reported, where values between −0.3 and +0.3 are treated as trivial associations. Hierarchical Linear Modeling (HLM) software version 7 (Raudenbush et al., 2011) was used to test multilevel logistic regression models of the effects of income inequality on ARPG. Multilevel statistical models are parametric models varying at more than one level. These are especially useful for research designs in which data are operationalized across more than one level (in the present study's case, individuals were nested within regions). Hierarchical linear models permit variance and covariance components to be partitioned across levels as well as the modeling of such variance by the inclusion of multilevel predictors (e.g., Molinaro et al., 2014; Vieno et al., 2015, 2016).

Due to the dichotomous nature of the dependent variable ARPG (yes/no), the models were analyzed with hierarchical generalized linear model (HGLM) using a Bernoulli sampling model with the following logit link function:

(1) $$\eta_{ijk}=log[\Phi_{ijk}/(1-\Phi_{ijk})]$$

where η _*ijk*_ is the log of the odds of being in the group reporting gambling and Φ_*ijk*_ is the probability of being member of this group. The initial analyses comprised an estimation of the unconditional model, where γ_00_ represented the average log-odds of being in the group of gamblers from the 21 Italian regions/cities taken into account. Next, the analysis involved simultaneously fitting two regression models for the dependent variable: a within-region model and a between-regions model. The within-region (Level 1), between individual-level variables and ARPG was examined (Model 1) for student i in region j, via the following equation:

η_ijk_ = π_0j_ + π_1j_(Male_ij_) + π _2j_(Family Wealth_ij_) + π _3j_(Other Family Types _ij_) + π _4j_ (Perceived Family support _ij_) + π _5j_(Perceived Peer support _ij_) + π _6j_(Perceived Teacher support._ij_) + π _7j_(Perceived Classmate support _ij_) + *e*_ij_

where η _*ij*_ is the log of the odds of being in the group of gamblers, π _0j_ is the intercept, π _1−7j_ are the parameters of the slopes for individual predictors, and *e*_ij_ is the level-1 error term. The between- region (Level 2) model estimated the influence of the GINI index and per capita GDP (at the regional level, Model 2) exerted on students' ARPG:

(2) $$\pi_{\text{0j}}=\beta_{00}+\beta_{01}(GINI_{\text{j}})+\beta_{02}(GDP_{\text{j}})+r_{0\text{j}}$$

Each of the Level-2 predictors were grand mean centered, and all the Level 1 slopes were controlled for their variations (i.e., free to have different effect across region).

## Results

**Figure 1:**
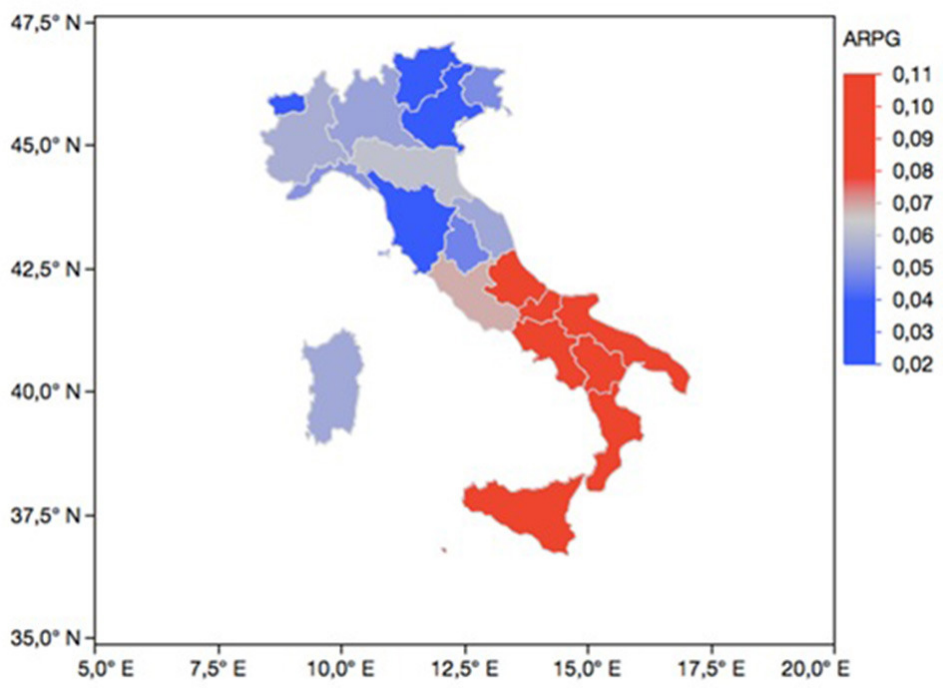
Regional prevalence of ARPG in 15-year-old Italian students (*n* = 20,791). ARPG (at-risk or problem gambling); Data reported for Trentino-Alto Adige region are the mean between two different geographic areas (Trento and Bolzano).

Among the nationally representative sample of Italian adolescents, the lifetime prevalence of gambling was 37.0%. Boys showed higher rates of lifetime gambling involvement compared to girls in each region (see Table 1). The reported levels of ARPG are 6.0% (total sample prevalence) with boys reporting higher levels of ARPG (10%) than girls (2%). In particular, the present study demonstrated a North–South gradient for the prevalence of ARPG, with higher prevalence of ARPG in the Southern/Islands/Central Regions (e.g., 11% in Sicily) than in Northern Italy (2% in Valle d'Aosta; Figure 1). With regard to gender distribution for each region, at-risk and problem gamblers were more likely to be male and less likely to be female (see Table 1).

Table 2 reports the means, standard deviations, and bivariate correlations for the individual and regional variables. With regard to the social support, students reported receiving more support from peers (*M* = 5.86; *SD* = 1.47) than from the family (*M* = 5.76; *SD* = 1.54); and from classmates (*M* = 3.78; *SD* = 0.81) than from teachers (*M* = 3.37; *SD* = 0.81). All bivariate correlations among study variables (at the individual and regional level) were in the hypothesized direction.

**Table 2:** Between individual- and regional-level variables: Descriptive statistics and correlations.

	**1**	**2**	**3**	**4**	**5**	**6**	**7**	**8**	**Mean (*SD*)**	**Minimum-maximum**
**INDIVIDUAL LEVEL (*N* = 20,791)**										
1. Gender (male)	–								0.50 (0.50)	0.0–1.0
2. Family Wealth	0.04^***^	–							2.01 (0.66)	1.0–3.0
3. Other Family Types	0.07^***^	−0.05^***^	–						0.29 (0.45)	0.0–1.0
4. Perceived Family support	0.12^***^	0.06^**^	−0.07^***^	–					5.76 (1.55)	1.0–7.0
5. Perceived Peer support	−0.08^***^	0.05^***^	−0.05^***^	0.30^***^	–				5.86 (1.47)	1.0–7.0
6. Perceived Teacher support	0.04^***^	−0.01	−0.04^***^	0.28^***^	0.123^***^	–			3.37 (0.81)	1.0–5.0
7. Perceived Classmate support	0.13^***^	0.04^***^	−0.03^***^	0.23^**^	0.29^***^	0.27^***^	–		3.78 (0.81)	1.0–5.0
8. ARPG	0.18^***^	−0.04^**^	0.06^***^	−0.05^***^	−0.05^**^	−0.04^***^	−0.02^**^	–	0.06 (0.23)	0.0–1.0
**REGIONAL LEVEL (*N* = 21)**^#^										
1. GINI	–								2.04 (0.74)	1.0–3.0
2. GDP per capita	−0.60^**^	–							26.26 (7.52)	16.20–39.90
3. ARPG	0.62^**^	−0.81^***^	–						0.06 (0.03)	0.01–0.11

*** p < 0.001;

** p < 0.01;

# *The number of regional levels totals 21 because the Trentino data comprised two different geographic areas (i.e., Bolzano and Trento)*.

The HLM models are reported in Table 3. The first step in HLM involved fitting an unconditional model (empty model) and comparing the empty model at one level (individuals) with the empty model at two levels (regions). The population-average estimate γ_00_ represented the average logs odds of ARPG in a region (γ_00_ = –2.812). This means that for a region with a random effect u_00_ = 0, the expected odds of being in the ARPG group was 0.094. Given the estimate of τ_00_ = 0.263 at the regional level, it was expected that 95% of the region would have log odds between −3.817 and −1.807, corresponding to a probability of reporting ARPG between 2.1 and 14.3%. The reliability for the unconditional model was 0.915 at the regional level.

**Table 3:** Odds ratios (95% CI) for reporting at-risk or problem gambling in relation to individual and regional variables.

	**Empty model**	**Model 1**	**Model 2**
**FIXED EFFECT**			
Intercept	0.06 (0.05–0.07)^***^	0.02 (0.01–0.03)^***^	0.02 (0.01–0.03)^***^
**INDIVIDUAL LEVEL (*N* = 20,791)**			
Males (reference = females)		6.43 (5.40–7.66)^***^	6.51 (5.44–7.80)^***^
Other family types (reference = Two biological or adoptive parents)		1.32 (1.15–1.51)^***^	1.33 (1.16–1.53)^***^
Family wealth medium-high (reference = low)		0.84 (0.76–0.93)^**^	0.85 (0.77–0.94)^***^
Perceived family support		0.87 (0.84–0.91)^***^	0.87 (0.83–0.92)^***^
Perceived peer support		0.97 (0.93–1.02)	0.97 (0.93–1.02)
Perceived teacher support		0.85 (0.78–0.92)^***^	0.85 (0.78–0.92)^**^
Perceived classmate support		0.94 (0.86–1.02)	0.94 (0.85–1.03)
**REGIONAL LEVEL (*****N*** = **21)**			
GDP per capita			0.95 (0.93–0.98)^***^
Gini			1.25 (1.06–1.47)^**^
**RANDOM EFFECT**			
Variance components	0.26 (0.51)	0.22 (0.47)	0.08 (0.26)
	$\chi_{\left(20\right)}^{2}$ = 239.87^***^	$\chi_{\left(20\right)}^{2}$ = 185.54^***^	$\chi_{\left(18\right)}^{2}$ = 66.40^***^

*** p < 0.001;

** p < 0.01;

*The number of regional levels totals 21 because the Trentino data comprised two different geographic areas (i.e., Bolzano and Trento)*.

The within-region model (Model 1) included individual variables. In the total sample model, males were more likely to be at-risk or problem gamblers. Adolescents not living with two biological or adoptive parents were significantly more likely to be ARPGs than adolescents living with two biological or adoptive parents. Additionally, adolescents who lived in more affluent families were significantly less likely to be ARPGs than those in a lower FAS family. With regard to social support variables, students who perceived more parental support reported less involvement in ARPG. Moreover, students who perceived stronger teacher support were less likely to be ARPGs. Finally, there were no associations between ARPG and perceived support from peers and classmates.

The between-region model (Model 2) included regional variables. In the 21 Italian regions/cities, income inequality (Gini index) was positively associated with ARPG. Thus, students who lived in a region/city with more pronounced income inequalities had higher odds of ARPG. Additionally, GDP per capita was negatively related to ARPG. Students who lived in a region/city in which GDP per capita was higher were less likely to be ARPGs. Additionally, in order to verify the possible different effects of perceived social support among adolescents living in different regions/cities, parallel analyses were performed by verifying the variability of these effects. A significant variability was only observed for perceived teacher support (*X*^2^ = 36.588, *p* < 0.05). However, none of the regional level predictors explained this variability.

## Discussion

The primary aim of the present study was to extend knowledge of adolescent gambling research by examining the association between structural determinants of adolescent gambling in a representative sample of adolescent students living in Italy. Three main results emerged from the data analysis. First, the results demonstrated that there was a North–South gradient for the prevalence of at-risk and problem gambling (ARPG) in Italy, with higher prevalence of at ARPG in the Southern/Islands/Central Regions (11% in Sicily/Abruzzo) than in Northern Italy (e.g., 2% in Valle d'Aosta). This result is partially consistent with previous reports showing that the prevalence of ARPG is higher for adolescents living in more disadvantaged regions in Italy (Gori et al., 2015). However, the present study provides, for the first time to the present authors' knowledge, demonstration of an association between income inequality and adolescent ARPG. More specifically, regional income inequalities (using GINI values) were positively related to ARPG. Thus, adolescent students who live in more unequal regions have a higher probability of being at-risk and problem gamblers (ARPGs). It is possible that larger income differences may increase gambling severity by increasing social status differences, status insecurities, competition and concerns about one's relative position in the social hierarchy (Wilkinson, 2004; Wilkinson and Pickett, 2009). These concerns start to become salient when adolescents are still developing a coherent understanding of social and economic hierarchies and their place in them (Yates and Youniss, 1999; Quintana, 1999). Adolescence is also a particularly sensitive developmental period characterized by a shift in the type of status that matters for adolescents, with their own money and position within the peer group begin to gain greater importance (i.e., adolescents start developing their own status positions).

According to risk sensitivity theory (i.e., Mishra et al., 2017), in more unequal regions, adolescents who experience disparities between one's present and desired outcomes would prefer relatively higher risk options, such as gambling. They may believe that in conditions of difficulty in satisfying a perceived need (i.e., money), greater risk-taking (e.g., involvement in gambling activities) is a way to satisfy such a need (Weber et al., 2004; Mishra and Fiddick, 2012). According to relative deprivation theories (Crosby, 1976; Walker and Smith, 2002), for such adolescents, gambling can be seen as a justice-seeking occupation (Callan et al., 2008) because gambling might offer resources to pursuing desirable outcomes (e.g., money, peer status) that adolescents might feel they merit but are otherwise unwilling or unable to reach via conventional means (e.g., having a job). In fact, gambling is seen as a means for monetary gain (Dechant and Ellery, 2011; Canale et al., 2015a; Devos et al., 2016), especially if traditional ways of making money are blocked and/or unavailable (Tabri et al., 2015). These potential explanations support contemporary theories of poverty, suggesting that what matters in affluent societies is the capacity to live life on a par with others (Sen, 1983; Townsend, 1979).

Additionally, adolescents living in more deprived contexts may be more prone to gamble because they believe that their self-worth is enhanced via gambling-related wins (Turner et al., 2002; Morasco et al., 2007) or may turn to gambling when they believe that economic mobility via traditional avenues is unlikely (Tabri et al., 2015). When this belief occurs, gambling can be used as a means to relieve their relative deprivation experience. Moreover, an additional explanation of why income inequality appears to increase ARPG might involve the disadvantage hypothesis, whereby stress arising from living in more unequal areas leads individuals to use substances as a coping mechanism (Caldwell et al., 2008). The distress that stems from living in unequal societies where individuals can feel angry and resentful when they believe that they have less than they deserve compared to others (for a review, see Smith et al., 2012), may drive them to gamble to cope with negative affect or to enhance positive affect. Such motivations are known to be positively associated with problem gambling in adolescents and young adults (e.g., Canale et al., 2015b; Lambe et al., 2015). Adolescents, like adults, may engage in potential risky behaviors (e.g., gambling and alcohol consumption) as a means to cope with feelings of deprivation and social disadvantage.

According to the conceptual framework of harmful gambling (Abbott et al., 2013), contextual macro-level factors such as gambling opportunities and macroeconomic indicators can help in explaining the potential effect of income inequality on ARPG. As gambling venues (in Australia, New Zealand and the United Kingdom) tend to be in areas of social deprivation (Wohl and Davis, 2017), being exposed to such a range of gambling opportunities may also foster pro-gambling attitudes (e.g., social approval and condoning of gambling), which in turn, could increase gambling involvement among adolescents. Additionally, income inequality may also be associated with lower government spending on public health services, thereby affecting the extent of exposure adolescents may have had to health promotion campaigns for reducing problem gambling.

Second, individuals who live in a region in which the GDP per capita is higher, have lower odds of being ARPGs. This finding supports neo-material theory (Lynch et al., 2000) in which higher availability of resources is associated with better health outcomes. It is possible to argue that wealthy regions have enough resources for health service provisions and benefits, such as expenditure on public health, which was been found to be associated with lower levels of probable gambling problems in representative samples of students living in nine European countries (Molinaro et al., 2014). Beyond income and wealth, differences in prevalence rates among regions may also be partially explained by large societal events, like natural disasters. In a study of risk related to natural disasters, increased risk-taking behavior was observed among disaster survivors (Norris et al., 2002; Vlahov et al., 2004) and perceived threat-to-life increases risk taking (Ben-Zur and Zeidner, 2009).

Third, the present study reported different results regarding the differential and unique impact of support sources on ARPG, that is, which source is more able to reduce the odds for adolescent to be at-risk and problematic gamblers. Consistent with results from previous studies (Hardoon et al., 2004; Räsänen et al., 2016; Canale et al., 2017), results from the main effects models indicated that adolescent students who perceived more support from parents and teachers reported less involvement in ARPG. It possible that positive relationships with parents and non-family adult mentors (e.g., teachers) foster feelings of safety in out-of-home settings among adolescents, and perceive the wider adult community as being supportive (e.g., Brooks et al., 2012), which in turn appears to have important preventive functions in inhibiting harmful forms of gambling. Additionally, results did not demonstrate that social support from peers and classmates accounted for ARPG. There are several observations that can be made regarding the absence of this effect. First, the study of the importance of social support sources indicated that friends and parents were perceived as equally supportive by 9- to 15-year-olds, but for those aged 16 to 18 years, the support of friends exceeded the support of parents (e.g., Bokhorst et al., 2010). Thus, it could be that the protective effect of peer support on gambling becomes more salient in older age adolescents. Consequently, future studies should include students from other school grades. Another explanation may be related to the fact that social motives (e.g., gambling to increase social affiliation) do not generally predict problem gambling in adolescents and young people (Stewart and Zack, 2008; Dechant and Ellery, 2011; Lambe et al., 2015).

Finally, contrary to what was hypothesized, no differences were found in perceived social support accounting for the association between income inequality and ARPG. According to the findings, the detrimental impact of regional inequality on adolescent gambling was the same for adolescents perceiving different levels of social support. However, other characteristics of the social environment (not considered in the current study) might amplify the impact of income inequality, such as the relevant dimensions of economic, social, and cultural capital that have been found to explain social inequality in adolescent health (e.g., food intake; De Clercq et al., 2016). The present study only considered social relationships that involved the adolescents' immediate social environment (i.e., the “microsystem” of family members, peer groups, classmates, and teachers) but social relationships outside this microsystem across neighborhoods, racial groups, and societies (more related to status competition) might moderate the association between income inequality and gambling (Putnam, 2000). For these reasons, future research is needed in order to explore other unconsidered factors related to income inequalities.

The present study is not without its limitations. First, the study utilized self-report data leading to well-known biases (such as memory recall biases and social desirability biases, etc.). Thus, the study depended upon adolescents' reports of and involvement in gambling and relationships (e.g., family wealth, social support). It would have been helpful to corroborate such self-reports with other informants such as parents or teachers. Second, the HBSC-Italy survey did not collect additional information on gambling behavior (e.g., gambling expenditure), nor was there any information about the types of gambling engaged in. Because previous studies have found that deprived areas present more gambling opportunities (e.g., in the form of gaming machines; Livingstone, 2001), future research should aim to explore the association between gambling frequency and income inequality. Third, a significant limitation of the present study was the cross-sectional design. Consequently, it cannot assume causality or rule out reverse causality. In fact, it is also possible that ARPG could lead to lower regional wealth or higher income inequality. For instance, the 2017 report of the Institute of Political, Economic and Social Studies (EURISPES, 2017) reported that gambling disorder represents the fourth leading cause of poverty in Italy. Examining these relationships longitudinally would provide a better understanding relating to the causal role of income inequality in the development of gambling problems among adolescents. Another limitation deriving from the cross-sectional nature of the data concerned the potential cumulative effect of inequality over time (e.g., McDonough et al., 2010). Future studies should focus on analyzing the differential effect that a different exposure to inequalities over time can have on adolescent gambling. Fourth, in accordance with the HBSC protocol, the participants were only 15-year-old students. Future studies should therefore investigate the association between income inequality and adolescent gambling severity with students from other school grades. Finally, the results of the present study cannot be generalized to 15-year-old students in other parts of the world where the socio-political structures may be very different.

Despite these limitations, to the best of the authors' knowledge, the present study is the first to investigate the effects of income inequality on ARPG in a large sample representative of the Italian high school population. In particular, the findings give support to the idea that adolescents who live in more unequal (and poor) regions show higher gambling-related harms. For this reason, policy actions are needed to redistribute wealth and create more egalitarian societies for reducing adolescent ARPG. Consequently, policy actions that concern limiting gambling need to support raising taxes on gambling, especially in a low gambling tax country like Italy. For example, Italy imposed tax rates on machines outside casinos up to four times lower than those imposed by Austria and Denmark (up to 13% in Italy compared to an average 55% in Denmark, and up to 50% in Austria [i.e., taxation of gambling services as a percentage of net revenue] (Sfetcu, 2016). Other policy recommendations to policymakers concerning adolescent problem gambling could include: (i) providing regions with more funding for implementing prevention programs at school level; (ii) limiting access to gambling opportunities (e.g., by imposing stricter penalties for gambling operators who allow minors to gamble illegally), and (iii) increasing public awareness (e.g., educating parents, teachers, and school administrators) that adolescents are not immune to gambling-related harms. In addition to redistributive fiscal policies aimed at promoting income equality at the regional level, our findings underline the need to implement prevention programs starting from more unequal regions, where prevention efforts are most needed. Educational interventions should also teach adolescents to recognize the attitudes and behaviors that discriminate, and reach out to adolescents in unequal areas for pro-responsible gambling policy. With regard to social support, the present study suggests that prevention efforts may benefit from being particularly mindful of those adolescents who lack social support from parents and teachers. Adolescents who perceived themselves as receiving less parental supervision are more likely to be at-risk and problem gamblers or frequent gamblers (e.g., Hardoon et al., 2004; Canale et al., 2016b). Thus, prevention programs could focus on teaching parents to develop trusting (and non-intrusive) parent–child relationships that foster honest self-disclosure. In conclusion, according to the adolescent risk behavior model incorporating youth gambling risk factors (Dickson et al., 2002 a model adapted from Jessor, 1998), the present study provides an example of how possible risk and protective factors operate in and across a number of domains (e.g., social environment and perceived environment). In conclusion, the present study for the first time (to our knowledge) was able to show that income inequality may have a contextual influence on ARPG. As this is the first demonstration of this association, substantial replication is required.

## Author contributions

NC designed the data collection instruments, conceptualized and designed the study, drafted the initial manuscript, and approved the final manuscript as submitted. AV carried out the initial analyses, designed the data collection instruments, and coordinated and supervised data collection, critically reviewed the manuscript, and approved the final manuscript as submitted. ML conceptualized and designed the study, designed the data collection instruments, reviewed and revised the manuscript, and approved the final manuscript as submitted. MG reviewed and revised various stages of the manuscript, and approved the final manuscript as submitted. AB designed the data collection instruments, and coordinated and supervised data collection, critically reviewed the manuscript, and approved the final manuscript as submitted. GL designed the data collection instruments, and coordinated and supervised data collection, critically reviewed the manuscript, and approved the final manuscript as submitted. PL designed the data collection instruments, and coordinated and supervised data collection, critically reviewed the manuscript, and approved the final manuscript as submitted. LS reviewed and revised the manuscript, and approved the final manuscript as submitted. MS reviewed and revised the manuscript, and approved the final manuscript as submitted. All authors approved the final manuscript as submitted and agree to be accountable for all aspects of the work.

### Conflict of interest statement

The authors declare that the research was conducted in the absence of any commercial or financial relationships that could be construed as a potential conflict of interest.

## Footnotes

^1^All Italian regions were involved, but the Trentino Region provided data for only two cities: Bolzano and Trento. Thus, the present study shows data for 19 regions (Abruzzo, Aosta Valley, Basilicata, Calabria, Campania, Emilia-Romagna, Friuli Venezia Giulia, Latium, Liguria, Lombardy, Marches, Molise, Piedmont, Puglia, Sicily, Sardinia, Tuscany, Umbria, and Veneto) and two cities (Bolzano and Trento).

## References

[B1] Abbott M. Binde P. Hodgins D. Korn D. Pereira A. Volberg R. (2013). Conceptual Framework of Harmful Gambling: An International Collaboration. Guelph, ON: Ontario Problem Gambling Research Centre.

[B2] American Psychiatric Association (2013). Diagnostic and Statistical Manual of Mental Disorders, 5th Edn. Washington, DC: American Psychiatric Association.

[B3] Barnes G. M. Welte J. W. Hoffman J. H. Dintcheff B. A. (1999). Gambling and alcohol use among youth: influences of demographic, socialization, and individual factors. Addict. Behav. 24, 749–767. 10.1016/S0306-4603(99)00048-9 10628510

[B4] Benhorin S. McMahon S. D. (2008). Exposure to violence and aggression: protective roles of social support among urban African American youth. J. Community Psychol. 36, 723–743. 10.1002/jcop.20252

[B5] Ben-Zur H. Zeidner M. (2009). Threat to life and risk-taking behaviors. A review of findings and explanatory models. Pers. Soc. Psychol. Rev. 13, 109–128. 10.1177/1088868308330104 19193927

[B6] Bjereld Y. Daneback K. Petzold M. (2017). Do bullied children have poor relationships with their parents and teachers? A cross-sectional study of Swedish children. Child. Youth Serv. Rev. 73, 347–351. 10.1016/j.childyouth.2017.01.012

[B7] Bokhorst C. L. Sumter S. R. Westenberg P. M. (2010). Social support from parents, friends, classmates, and teachers in children and adolescents aged 9 to 18 years: who is perceived as most supportive? Soc. Dev. 19, 417–426. 10.1111/j.1467-9507.2009.00540.x

[B8] Boyce W. Torsheim T. Currie C. Zambon A. (2006). The family affluence scale as a measure of national wealth: validation of an adolescent self-report measure. Soc. Indic. Res. 78, 473–487. 10.1007/s11205-005-1607-6

[B9] Brooks F. M. Magnusson J. Spencer N. Morgan A. (2012). Adolescent multiple risk behaviour: an asset approach to the role of family, school and community. J. Public Health 34, i48–i56. 10.1093/pubmed/fds001 22363031

[B10] Calado F. Alexandre J. Griffiths M. D. (2016). Prevalence of adolescent problem gambling: A systematic review of recent research. J. Gambl. Stud. 33, 397–424. 10.1007/s10899-016-9627-5 27372832 PMC5445143

[B11] Caldwell T. M. Rodgers B. Clark C. Jefferis B. J. M. H. Stansfeld S. A. Power C. (2008). Lifecourse socioeconomic predictors of midlife drinking patterns, problems and abstention: findings from the 1958 British Birth Cohort study. Drug Alcohol Depend. 95, 269–278. 10.1016/j.drugalcdep.2008.01.014 18339490

[B12] Callan M. J. Ellard J. H. Shead N. W. Hodgins D. C. (2008). Gambling as a search for justice: examining the role of personal relative deprivation in gambling urges and gambling behavior. Pers. Soc. Psychol. Bull. 34, 1514–1529. 10.1177/0146167208322956 18723773

[B13] Callan M. J. Shead N. W. Olson J. M. (2015). The relation between personal relative deprivation and the urge to gamble among gamblers is moderated by problem gambling severity: a meta-analysis. Addict. Behav. 45, 146–149. 10.1016/j.addbeh.2015.01.031 25665918

[B14] Canale N. Griffiths M. D. Vieno A. Siciliano V. Molinaro S. (2016a). Impact of Internet gambling on problem gambling among adolescents in Italy: findings from a large-scale nationally representative survey. Comput. Hum. Behav. 57, 99–106. 10.1016/j.chb.2015.12.020

[B15] Canale N. Santinello M. Griffiths M. D. (2015a). Validation of the reasons for gambling questionnaire (RGQ) in a British population survey. Addict. Behav. 45, 276–280. 10.1016/j.addbeh.2015.01.035 25746361

[B16] Canale N. Vieno A. Griffiths M. D. Borraccino A. Lazzeri G. Charrier L.. (2017). A large-scale national study of gambling severity among immigrant and non-immigrant adolescents: the role of the family. Addict. Behav. 66, 125–131. 10.1016/j.addbeh.2016.11.020 27930902

[B17] Canale N. Vieno A. Griffiths M. D. Rubaltelli E. Santinello M. (2015b). How do impulsivity traits influence problem gambling through gambling motives? The role of perceived gambling risk/benefits. Psychol. Addict. Behav. 29, 813–823. 10.1037/adb0000060 25730629

[B18] Canale N. Vieno A. Ter Bogt T. Pastore M. Siciliano V. Molinaro S. (2016b). Adolescent gambling-oriented attitudes mediate the relationship between perceived parental knowledge and adolescent gambling: implications for prevention. Prev. Sci. 17, 970–980. 10.1007/s11121-016-0683-y 27448214

[B19] Caraco T. Martindale S. Whittam T. S. (1980). An empirical demonstration of risk-sensitive foraging preferences. Anim. Behav. 28, 820–830. 10.1016/S0003-3472(80)80142-4

[B20] Chiesi F. Donati M. A. Galli S. Primi C. (2013). The suitability of the South Oaks Gambling Screen–Revised for Adolescents (SOGS-RA) as a screening tool: IRT-based evidence. Psychol. Addict. Behav. 27:287. 10.1037/a0029987 23025708

[B21] Compas B. E. Hinden B. R. Gerhardt C. A. (1995). Adolescent development: pathways and processes of risk and resilience. Annu. Rev. Psychol. 46, 265–293. 10.1146/annurev.ps.46.020195.001405 7872731

[B22] Crosby F. (1976). A model of egoistical relative deprivation. Psychol. Rev. 83, 85–113. 10.1037/0033-295X.83.2.85

[B23] Currie C. Molcho M. Boyce W. Holstein B. Torsheim T. Richter M. (2008). Researching health inequalities in adolescents: the development of the Health Behaviour in School-Aged Children (HBSC) family affluence scale. Soc. Sci. Med. 66, 1429–1436. 10.1016/j.socscimed.2007.11.024 18179852

[B24] De Clercq B. Abel T. Moor I. Elgar F. J. Lievens J. Sioen I. (2016). Social inequality in adolescents' healthy food intake: the interplay between economic, social and cultural capital. Eur. J. Public Health 15:203 10.1093/eurpub/ckw236 28040734

[B25] Dechant K. Ellery M. (2011). The effect of including a monetary motive item on the gambling motives questionnaire in a sample of moderate gamblers. J. Gambl. Stud. 27, 331–344. 10.1007/s10899-010-9197-x 20496161 PMC3102211

[B26] Demaray M. K. Malecki C. K. (2002). The relationship between perceived social support and maladjustment for students at risk. Psychol. Schools 39, 305–316. 10.1002/pits.10018

[B27] Devos G. Challet-Bouju G. Burnay J. Maurage P. Grall-Bronnec M. Billieux J. (2016). Adaptation and validation of the Gambling Motives Questionnaire-Financial (GMQ-F) in a sample of French-speaking gamblers. Int. Gambl. Stud. 17, 87–101. 10.1080/14459795.2016.1264080

[B28] Dickson L. M. Derevensky J. L. Gupta R. (2002). The prevention of gambling problems in youth: a conceptual framework. J. Gambl. Stud. 18, 97–159. 10.1023/A:1015557115049 12096450

[B29] Dorling D. Mitchell R. Pearce J. (2007). The global impact of income inequality on health by age: an observational study. BMJ 335:873. 10.1136/bmj.39349.507315.DE 17954512 PMC2043415

[B30] Edgren R. Castrén S. Mäkelä M. Pörtfors P. Alho H. Salonen A. H. (2016). Reliability of instruments measuring at-risk and problem gambling among young individuals: a systematic review covering years 2009–2015. J. Adolesc. Health 58, 600–615. 10.1016/j.jadohealth.2016.03.007 27151759

[B31] Elgar F. J. Craig W. Boyce W. Morgan A. Vella-Zarb R. (2009). Income inequality and school bullying: multilevel study of adolescents in 37 countries. J. Adolesc. Health 45, 351–359. 10.1016/j.jadohealth.2009.04.004 19766939

[B32] Elgar F. J. Pförtner T. K. Moor I. De Clercq B. Stevens G. W. Currie C. (2015). Socioeconomic inequalities in adolescent health 2002–2010: a time-series analysis of 34 countries participating in the Health Behaviour in School-aged Children study. Lancet 385, 2088–2095. 10.1016/S0140-6736(14)61460-4 25659283

[B33] Elgar F. J. Roberts C. Parry-Langdon N. Boyce W. (2005). Income inequality and alcohol use: a multilevel analysis of drinking and drunkenness in adolescents in 34 countries. Eur. J. Public Health 15, 245–250. 10.1093/eurpub/cki093 15985459

[B34] EURISPES (2017). Rapporto Italia 2017. Si sente Povero un Italiano su Quattro. Available online at: http://www.eurispes.eu/content/eurispes-rapporto-italia-2017-si-sente-povero-un-italiano-su-quattro (Accessed February 23, 2017).

[B35] Gori M. Potente R. Pitino A. Scalese M. Bastiani L. Molinaro S. (2015). Relationship between gambling severity and attitudes in adolescents: findings from a population-based study. J. Gambl. Stud. 31, 717–740. 10.1007/s10899-014-9481-2 25063468

[B36] Haisley E. Mostafa R. Loewenstein G. (2008). Subjective relative income and lottery ticket purchases. J. Behav. Decis. Mak. 21, 283–295. 10.1002/bdm.588

[B37] Hamilton H. A. van der Maas M. Boak A. Mann R. E. (2014). Subjective social status, immigrant generation, and cannabis and alcohol use among adolescents. J. Youth Adolesc. 43, 1163–1175. 10.1007/s10964-013-0054-y 24218067

[B38] Hardoon K. K. Gupta R. Derevensky J. L. (2004). Psychosocial variables associated with adolescent gambling. Psychol. Addict. Behav. 18, 170–179. 10.1037/0893-164X.18.2.170 15238059

[B39] Jessor R. (1998). New perspectives on adolescent risk behavior, in New Perspectives on Adolescent Risk Behavior, ed Jessor R. (Cambridge: Cambridge University Press), 1–12.

[B40] Jessor R. Turbin M. S. Costa F. M. Dong Q. Zhang H. Wang C. (2003). Adolescent problem behavior in China and the United States: a cross-national study of psychosocial protective factors. J. Res. Adolesc. 13, 329–360. 10.1111/1532-7795.1303004

[B41] Juang L. P. Silbereisen R. K. (1999). Supportive parenting and adolescent adjustment across time in former East and West Germany. J. Adolesc. 22, 719–736. 10579886 10.1006/jado.1999.0267

[B42] Klemera E. Brooks F. M. Chester K. L. Magnusson J. Spencer N. (2016). Self-harm in adolescence: protective health assets in the family, school and community. Int. J. Public Health 62, 631–638. 10.1007/s00038-016-0900-2 27658811 PMC5487889

[B43] Lambe L. Mackinnon S. P. Stewart S. H. (2015). Validation of the gambling motives questionnaire in emerging adults. J. Gambl. Stud. 31, 867–885. 10.1007/s10899-014-9467-0 24871297

[B44] Lazzeri G. Giacchi M. V. Dalmasso P. Vieno A. Nardone P. Lamberti A.. (2013). The methodology of the Italian HBSC 2010 study (Health Behaviour in School-aged Children). Ann. Ig. 25, 225–233. 10.7416/ai.2013.1925 23598806

[B45] Livingstone C. (2001). The social economy of poker machine gambling in Victoria. Int. Gambl. Stud. 1, 46–65. 10.1080/14459800108732287

[B46] Lynch J. DUE P. Muntaner C. Smith G. D. (2000). Social capital—is it a good investment strategy for public health? J. Epidemiol. Commun. Health 54, 404–408. 10818113 10.1136/jech.54.6.404 PMC1731686

[B47] McDonough P. Worts D. Sacker A. (2010). Socioeconomic inequalities in health dynamics: a comparison of Britain and the United States. Soc. Sci. Med. 70, 251–260. 10.1016/j.socscimed.2009.10.001 19857919

[B48] Messerlian C. Derevensky J. L. (2005). Youth gambling: a public health perspective. Health Promot. Int. 20, 69–79. 10.1093/heapro/dah509 15681591

[B52] Mishra S. Barclay P. Lalumière M. L. (2014). Competitive disadvantage facilitates risk taking. Evol. Hum. Behav. 35, 126–132. 10.1016/j.evolhumbehav.2013.11.006

[B49] Mishra S. Barclay P. Sparks A. (2017). The relative state model: integrating need-based and ability-based pathways to risk-taking. Pers. Soc. Psychol. Rev. 21, 176–198. 10.1177/1088868316644094 27149981

[B50] Mishra S. Fiddick L. (2012). Beyond gains and losses: the effect of need on risky choice in framed decisions. J. Pers. Soc. Psychol. 102, 1136–1147. 10.1037/a0027855 22486678

[B51] Mishra S. Novakowski D. (2016). Personal relative deprivation and risk: an examination of individual differences in personality, attitudes, and behavioral outcomes. Pers. Individ. Dif. 90, 22–26. 10.1016/j.paid.2015.10.031

[B53] Molinaro S. Canale N. Vieno A. Lenzi M. Siciliano V. Gori M.. (2014). Country–and individual–level determinants of probable problematic gambling in adolescence: a multi–level cross–national comparison. Addiction 109, 2089–2097. 10.1111/add.12719 25171305

[B54] Moor I. Rathmann K. Lenzi M. Pförtner T. K. Nagelhout G. E. de Looze M.. (2015). Socioeconomic inequalities in adolescent smoking across 35 countries: a multilevel analysis of the role of family, school and peers. Eur. J. Public Health 25, 457–463. 10.1093/eurpub/cku244 25713016

[B55] Morasco B. J. Weinstock J. Ledgerwood D. M. Petry N. M. (2007). Psychological factors that promote and inhibit pathological gambling. Cogn. Behav. Pract. 14, 208–217. 10.1016/j.cbpra.2006.02.005

[B56] Ng Fat L. Scholes S. Jivraj S. (2016). The relationship between drinking pattern, social capital, and area-deprivation: findings from the Health Survey for England. J. Stud. Alcohol Drugs 78, 20–29. 10.15288/jsad.2017.78.20 27936361

[B57] Norris F. H. Friedman M. J. Watson P. J. Byrne C. M. Diaz E. Kaniasty K. (2002). 60,000 disaster victims speak: part, I. An empirical review of the empirical literature, 1981-2001. Psychiatry 65, 207–239. 10.1521/psyc.65.3.207.20173 12405079

[B58] Orford J. Wardle H. Griffiths M. Sproston K. Erens B. (2010). PGSI and DSM-IV in the 2007 British gambling prevalence survey: reliability, item response, factor structure and inter-scale agreement. Int. Gambl. Stud. 10, 31–44. 10.1080/14459790903567132

[B59] Pickett K. E. Wilkinson R. G. (2015). Income inequality and health: a causal review. Soc. Sci. Med. 128, 316–326. 10.1016/j.socscimed.2014.12.031 25577953

[B60] Potenza M. N. Wareham J. D. Steinberg M. A. Rugle L. Cavallo D. A. Krishnan-Sarin S.. (2011). Correlates of at-risk/problem internet gambling in adolescents. J. Am. Acad. Child Adolesc. Psychiatry 50, 150–159. 10.1016/j.jaac.2010.11.006 21241952 PMC3190180

[B61] Putnam R. D. (2000). Bowling Alone: Collapse and Revival of American community. New York, NY: Simon and Schuster.

[B62] Quintana S. M. (1999). Children's developmental understanding of ethnicity and race. Appl. Prev. Psychol. 7, 27–45. 10.1016/S0962-1849(98)80020-6

[B63] Räsänen T. Lintonen T. Tolvanen A. Konu A. (2016). Social support as a mediator between problem behaviour and gambling: a cross-sectional study among 14–16-year-old Finnish adolescents. BMJ Open 6:e012468. 10.1136/bmjopen-2016-012468 28007707 PMC5223705

[B64] Raudenbush S. W. Bryk A. S. Cheong Y. F. Congdon R. Du Toit M. (2011). Hierarchical Linear and Nonlinear Modeling (HLM7). Lincolnwood, IL: Scientific Software International.

[B65] Reith G. (2012). Beyond addiction or compulsion: the continuing role of environment in the case of pathological gambling. Addiction 107, 1736–1737. 10.1111/j.1360-0443.2012.03669.x 22962952

[B66] Reith G. Dobbie F. (2011). Beginning gambling: the role of social networks and environment. Addict. Res. Theory 19, 483–493. 10.3109/16066359.2011.558955

[B67] Saunders J. Davis L. Williams T. Williams J. H. (2004). Gender differences in self-perceptions and academic outcomes: a study of African American high school students. J. Youth Adolesc. 33, 81–90. 10.1023/A:1027390531768

[B68] Sen A. (1983). Poor, relatively speaking. Oxf. Econ. Pap. 35, 153–169. 10.1093/oxfordjournals.oep.a041587

[B69] Sfetcu N. (2016). Gaming Guide: Gambling in Europe. Createspace Independent Publishing Platform.

[B70] Smith H. J. Pettigrew T. F. Pippin G. M. Bialosiewicz S. (2012). Relative deprivation: a theoretical and meta-analytic review. Pers. Soc. Psychol. Rev. 16, 203–232. 10.1177/1088868311430825 22194251

[B71] Stewart S. H. Zack M. (2008). Development and psychometric evaluation of a three-dimensional Gambling Motives Questionnaire. Addiction 103, 1110–1117. 10.1111/j.1360-0443.2008.02235.x 18554344

[B72] Tabri N. Dupuis D. R. Kim H. S. Wohl M. J. (2015). Economic mobility moderates the effect of relative deprivation on financial gambling motives and disordered gambling. Int. Gambl. Stud. 15, 309–323. 10.1080/14459795.2015.1046468

[B73] Tabri N. Shead N. W. Wohl M. J. (2017). Me, Myself, and Money II: Relative deprivation predicts disordered gambling severity via delay discounting, especially among gamblers who have a financially focused self-concept. J. Gambl. Stud. [Epub ahead of print]. 10.1007/s10899-017-9673-7 28154956

[B74] The World Top Incomes Database (2011). Available online at: http://www.parisschoolofeconomics.eu/en/news/the-top-incomes-database-new-website/ (Accessed February 23, 2017).

[B75] Thewissen S. H. Kenworthy L. Nolan B. Roser M. Smeeding T. (2015). Rising Income Inequality and Living Standards in OECD Countries: How does the Middle Fare? LIS Working Paper Series. Oxford: Institute for New Economic Thinking, Oxford Martin School.

[B76] Torsheim T. Wold B. Samdal O. (2000). The teacher and classmate support scale: factor structure, test-retest reliability and validity in samples of 13-and 15-year-old adolescents. Sch. Psychol. Int. 21, 195–212. 10.1177/0143034300212006

[B77] Townsend P. (1979). Poverty in the United Kingdom: A Survey of Household Resources and Standards of Living. Berkeley, CA: University of California Press.

[B78] Turner N. E. Zangeneh M. Littman Sharp N. Spence W. (2002). Why do Some Develop Gambling Problems While Others Do Not? Report prepared for the Ontario Ministry of Health and Long Term Care. Guelph, ON: Ontario Problem Gambling Centre.

[B79] Vieno A. Gini G. Lenzi M. Pozzoli T. Canale N. Santinello M. (2015). Cybervictimization and somatic and psychological symptoms among Italian middle school students. Eur. J. Public Health 25, 433–437. 10.1093/eurpub/cku191 25465914

[B80] Vieno A. Lenzi M. Roccato M. Russo S. Monaci M. G. Scacchi L. (2016). Social capital and fear of crime in adolescence: a multilevel study. Am. J. Commun. Psychol. 58, 100–110. 10.1002/ajcp.12071 27435954

[B81] Viner R. M. Ozer E. M. Denny S. Marmot M. Resnick M. Fatusi A.. (2012). Adolescence and the social determinants of health. Lancet 379, 1641–1652. 10.1016/S0140-6736(12)60149-4 22538179

[B82] Vlahov D. Galea S. Ahern J. Resnick H. Kilpatrick D. (2004). Sustained increased consumption of cigarettes, alcohol, and marijuana among Manhattan residents after September 11, 2001. J. Public Health 94, 253–254. 10.2105/AJPH.94.2.253 14759935 PMC1448236

[B83] Volberg R. A. Gupta R. Griffiths M. D. Ólason D. T. Delfabbro P. (2010). An international perspective on youth gambling prevalence studies. Int. J. Adolesc. Med. Health, 22, 3–38. 10.1515/IJAMH.2010.22.1.3 20491416

[B84] Walker I. Smith H. J. (eds.). (2002). Relative Deprivation: Specification, Development, and Integration. New York, NY: Cambridge University Press.

[B85] Wardle H. Keily R. Astbury G. Reith G. (2014). ‘Risky places?’: mapping gambling machine density and socio-economic deprivation. J. Gambl. Stud. 30, 201–212. 10.1007/s10899-012-9349-2 23242474

[B86] Weber E. U. Shafir S. Blais A. R. (2004). Predicting risk sensitivity in humans and lower animals: risk as variance or coefficient of variation. Psychol. Rev. 111:430 10.1037/0033-295X.111.2.430 15065916

[B87] Wickwire E. M. Whelan J. P. Meyers A. W. Murray D. M. (2007). Environmental correlates of gambling behavior in urban adolescents. J. Abnorm. Child Psychol. 35, 179–190. 10.1007/s10802-006-9065-4 17219080

[B88] Wilkinson R. (2004). Why is violence more common where inequality is greater? Ann. N. Y. Acad. Sci. 1036, 1–12. 10.1196/annals.1330.001 15817728

[B89] Wilkinson R. G. Pickett K. E. (2009). Income inequality and social dysfunction. Annu. Rev. Sociol. 35, 493–511. 10.1146/annurev-soc-070308-115926

[B90] Wills T. A. Shinar O. (2000). Measuring perceived and received social support, in Social Support Measurement and Intervention: A Guide for Health and Social Scientists, eds Cohen S. Underwood L. G. Gottlieb B. H. (Oxford: Oxford University Press), 86–135.

[B91] Winters K. C. Stinchfield R. D. Kim L. G. (1995). Monitoring adolescent gambling in Minnesota. J. Gambl. Stud. 11, 165–183. 10.1007/BF02107113 24233428

[B92] Wohl M. J. Davis C. G. (2017). Finding some straw in the ‘Industry–State Gambling Complex’ argument: commentary on Delfabbro and King. Int. Gambl. Stud. [Epub ahead of print]. 10.1080/14459795.2017.1312483

[B93] Yates M. Youniss J. (1999). Roots of Civic Identity: International Perspectives on Community Service and Activism in Youth. Cambridge: Cambridge University Press.

[B94] Zimet G. D. Dahlem N. W. Zimet S. G. Farley G. K. (1988). The multidimensional scale of perceived social support. J. Pers. Assess. 52, 30–41. 10.1207/s15327752jpa5201_2 2280326

